# Species abundance and temporal variation of arbovirus vectors in Brownsville, Texas

**DOI:** 10.26633/RPSP.2017.28

**Published:** 2017-03-23

**Authors:** Krithika Srinivasan, Beatriz Tapia, Arturo Rodriguez, Robert Wood, Jennifer J Salinas

**Affiliations:** 1 University of Texas Health Science Center at San Antonio University of Texas Health Science Center at San Antonio San AntonioTexas United States of America University of Texas Health Science Center at San Antonio, San Antonio, Texas, United States of America.; 2 City of Brownsville Department of Public Health City of Brownsville Department of Public Health BrownsvilleTexas United States of America City of Brownsville Department of Public Health, Brownsville, Texas, United States of America.; 3 Texas Tech University Health Sciences Center at El Paso Texas Tech University Health Sciences Center at El Paso El PasoTexas United States of America Texas Tech University Health Sciences Center at El Paso, El Paso, Texas, United States of America.

**Keywords:** Arboviruses, vector control, *Aedes*, border health, health surveillance, Texas, Mexico, United States, Americas, Arbovirus, control de vectores, *Aedes*, salud fronteriza, vigilancia sanitaria, Texas, México, Estados Unidos, Américas

## Abstract

The recent outbreaks of the dengue fever and West Nile viruses and the looming threats of the Zika and chikungunya viruses highlight the importance of establishing effective, proactive arboviral surveillance in communities at high risk of transmission, such as those on the Texas–Mexico border. Currently, there are no approved human vaccines available for these mosquito-borne diseases, so entomological control and case management are the only known methods for decreasing disease incidence. The principal vectors, which include Culex quinquefasciatus, Aedes aegypti, and Ae. Albopictus, all have an established presence in South Texas. The public health response to most arbovirus outbreaks in the region has been reactionary rather than proactive. However, after the 2005 dengue outbreak and subsequent fatality, the City of Brownsville Public Health Department began collecting data on mosquito vector abundance and incidence. The objective of this study was to describe the various species of mosquitoes found in vector surveillance in Brownsville, Texas, during 2009–2013; quantify their prevalence; and identify any associations with temporal or weather-related variations. The results confirm a significant mosquito population in Brownsville in late winter months, indicating a high risk of arbovirus transmission in South Texas year-round, and not just until November, previously considered the end date of arbovirus season by state health services. The data from Brownsville’s surveillance program can help characterize local vector ecology and facilitate more proactive mitigation of future arboviral threats in South Texas.

## RESUMEN

Los últimos brotes de los virus del dengue y del Nilo Occidental y la inminente amenaza del virus del Zika y el chikunguña ponen de relieve la importancia de instaurar un sistema eficaz y proactivo de vigilancia de los arbovirus en las comunidades expuestas a un riesgo alto de transmisión, como las ubicadas en la frontera entre Texas y México. Actualmente no se dispone de ninguna vacuna humana aprobada contra estas enfermedades transmitidas por mosquitos, de manera que el control entomológico y el manejo de los casos son los únicos métodos conocidos para reducir la incidencia de estas enfermedades. Está comprobada la presencia de los princi-pales vectores, entre los que se cuentan las especies *Culex quinquefasciatus, Aedes aegypti* y *Ae. albopictus,* en la zona sur de Texas. La respuesta de salud pública a la mayoría de los brotes de arbovirus en la región ha sido reactiva en lugar de proactiva. Sin embargo, después del brote de dengue registrado en el 2005 y dada su letalidad, el Departamento de Salud Pública de la Ciudad de Brownsville empezó a recopilar datos sobre la abundancia de los mosquitos vectores y la incidencia de las enfermedades que transmi-ten. El objetivo de este estudio fue describir las diversas especies de mos-quitos encontrados mediante la vigilancia de vectores realizada en Brownsville (Texas) entre los años 2009 y 2013, cuantificar su prevalencia y determinar si hay alguna relación con las variaciones temporales o climáti-cas. Los resultados confirman que Brownsville tiene una población signifi-cativa de mosquitos en los últimos meses del invierno, lo que indica un alto riesgo de transmisión de arbovirus en la zona sur de Texas durante todo el año y no solo hasta noviembre, mes en el que los servicios de salud del estado solían considerar que culminaba la temporada de arbovirus. Los datos del programa de vigilancia de Brownsville pueden ayudar a caracte-rizar la ecología local de los vectores y propiciar una mitigación más proac-tiva de las futuras amenazas arbovirales en la zona sur de Texas.

In Texas, arboviral surveillance systems were first initiated to mitigate outbreaks of West Nile virus (WNV). The valuable information this type of surveillance could generate about recent arboviral threats such as dengue fever virus (DENV), chikungunya virus (CHIKV), Saint Louis encephalitis virus (SLEV), and Zika virus (ZIKV) could significantly affect morbidity and mortality in local communities. This information is particularly timely as the Texas Department of State Health Services (TDSHS) reported the first case of locally acquired mosquito-borne ZIKV infection in the city of Brownsville, Texas, on November 28, 2016. Subsequently, on December 9, 2016, four additional cases were acquired, suggesting the possibility of local transmission of ZIKV in Texas (1).

Brownsville and South Texas are not strangers to arboviral threats. While DENV has historically been present in South Texas, a yellow fever virus (YFV) campaign conducted in the 1940s largely exterminated its *Aedes* vectors (*Ae. aegypti* and *Ae. Albopictus*). However, since the 1980s there has been a resurgence in the virus and its vectors (2, 3). In a 1980 outbreak in South Texas, one of the largest ever, 25 locally acquired cases and 27 imported cases were recorded, for a total of 52 reported cases of DENV. Since then additional outbreaks have been documented with increasing frequency. Peak activity occurred in 1999, with six local and 14 imported cases, and in 2005, with three local and 23 imported cases (4). In 2013, South Texas experienced another significant DENV outbreak, with 53 laboratory-confirmed cases. Twenty- six of those cases reported no recent travel, making the outbreak the largest number of clinical, locally acquired cases reported during a single year (5). More than half of all cases were identified in Cameron County, in the border city of Brownsville, Texas. This was unsurprising, given Brownsville’s close proximity to Mexico, which was also experiencing a DENV outbreak at the same time.

## SURVEILLANCE AND VECTOR CONTROL STRATEGIES

Brownsville has long been regarded as one of the most vulnerable regions in the United States for outbreaks of mosquito-borne diseases (6, 7). With its favorable vector ecology (e.g., environmental effects on vector incidence), this and other areas in South Texas are considered highrisk for arbovirus activity, particularly DENV (8, 9). Some of this risk is attributed to the humid, subtropical, coastal climate that results in year-round temperatures ideal for vector breeding (10). In Brownsville, the average maximum temperature from 1981 to 2010 was 83 degrees and the average minimum temperature was 65.4 degrees. Currently, there are no commercially available human vaccines or targeted drug therapies for CHIKV, DENV, WNV, or ZIKV, leaving mosquito control as the principal method of disease prevention (11–13). Yet very little research has been carried out on vector species’ habitat. In the Texas–Mexico border region, most U.S.-based research has focused on identifying cases, investigating peri-domicilar areas for vector breeding containers, and establishing the likelihood of autochthonous versus travel-acquired DENV (2, 14).

Directly across the border from Brownsville, in Matamoros, Mexico, similar efforts are under way. It has long been understood that the Mexican side of the border has a significantly higher burden of arboviral disease than the U.S. side, even though the two entities are only divided by the Rio Grande River (15). Therefore, Mexico devotes a great amount of resources to proactive human case identification, and mosquito extermination, using chemicals and ovi-traps, rather than monitoring species abundance and incidence with gravid traps, like the surveillance program analyzed by this study. Regardless of the methods used, effective vector control requires a good understanding of vector ecology. The objective of this study was to describe the various species of mosquitoes found by vector surveillance in Brownsville, Texas, during 2009–2013; quantify their prevalence; and identify any associations with temporal or weather-related variations. This study is the first in a series of steps to examine environmental factors that potentially drive the transmission of arboviruses in the South Texas region.

### Collection and testing methods

Systematic collection and testing of mosquitoes leads to better understanding of vector ecology. Therefore, the City of Brownsville Public Health Department has used a vector control and surveillance strategy since 2009. Starting in June and continuing through November, gravid traps from the U.S. Centers for Disease Control and Prevention (CDC) are placed around the city twice per week, in predetermined locations, and retrieved within 24 hours. Traps are also placed in response to community complaints. The mosquitoes are collected from the traps, transferred, alive, into a container, and shipped to TDSHS in Austin for species identification, pooling, and virus testing. Female mosquitoes are separated by species, and only those known to be human disease vectors are pooled for testing. After mosquito samples are homogenized and centrifuged, supernatants are inoculated onto two vertebrate cell lines and observed for 10 days. If cytopathic effects (CPEs) are observed, virus identification tests are performed using an indirect fluorescent antibody (IFA) assay for Western equine encephalitis virus (WEEV), Eastern equine encephalitis virus (EEEV), Venezuelan equine encephalitis (VEEV), SLEV, California encephalitis virus (CEV), Highlands J virus (HJV), Tensaw virus (TV), and CHIKV. If the results are inconclusive, samples are sent to the CDC Arbovirus Diagnostic Laboratory in Fort Collins, Colorado, for further identification (16).

Using arbovirus isolation and mosquito species data from TDSHS and the local National Oceanic and Atmospheric Administration (NOAA) weather station, the Brownsville Public Health Department surveillance teams gained a better understanding of local vector ecology. First, they looked at mosquito species abundance in the region. Although the presence of *Ae. aegypti* and *Ae. albopictus* in South Texas had already been established, they wanted a better understanding of other potential disease vectors (9). A wide variety of mosquito species were caught in the surveillance traps and identified. Subsequent studies focused on the three most common species and any associations between their incidence and temporal or weather variations in Brownsville.

## RESULTS

### Species abundance and incidence

Table 1 shows a list of all species isolated in Brownsville from 2009 to 2013. Of the 23 different species caught in the gravid traps, the most common were *Culex quinquefasciatus* (82%), *Ae. aegypti* (16%), *Ae. albopictus* (1%), and “other” (> 1%). Vector abundance was examined by species and by month to evaluate vector species dynamics throughout the arbovirus season—an important factor in optimizing resource allocation. Because so few *Ae. albopictus* mosquitoes were found, the category for that species was combined with the *Ae. aegypti* group under a new name (*“Aedes* spp.”) for analysis of species incidence over time (Table 2). The data showed a large decrease in the percentage of *C. quinquefasciatus* from 2009 to 2010, followed by a gradual increase through 2013.

**TABLE 1.** Mosquito species collected in an arbovirus vector surveillance program using gravid traps, Brownsville, Texas, 2009–2013

Aedes aegypti Aedes albopictus Aedes bimaculatus Aedes epacitus Aedes sollicitans Aedes taeniorhynchus Anopheles pseudopunctipennis Anopheles punctipennis *Anopheles* spp. Anopheles crucians Anopheles quadrimaculatus Culex coronator *Culex (Melanoconion)* spp. Culex nigripalpus Culex quinquefasciatus Culex salinarius Culiseta inornata Deinocerites mathesoni Mansonia titillans Toxorhynchites rutilus septentrionalis *Uranotaenia* spp.

***Source:*** Prepared by the authors based on the study results.

**TABLE 2.** Weather statistics and arbovirus mosquito vector species incidence by year based on pooled data from gravid traps (*n* = 35), Brownsville, Texas, 2009–2013

Variable	2009		2010		2011		2012		2013	
Average rainfall per month (inches) (SD^a^)	2.9	(4.4)	3.9	(4.4)	1.7	(3.0)	1.3	(1.1)	0.95	(0.902)
Average temperature highs (°F) (SD)	86.6	(11.2)	89.3	(5.2)	90.9	(4.8)	91.9	(3.0)	91.5	(3.6)
Proportion (%) of insect trap collections by species^b^ (SD)										
Culex q.	95.1	(5.0)	76.4	(25.8)	76.6	(19.4)	80.4	(14.5)	80.8	(20.7)
*Aedes* spp.	4.7	(4.8)	23.0	(25.6)	23.0	(19.8)	22.0	(11.3)	17.6	(21.2)
Ae. aegypti	4.6	(4.7)	21.4	(25.7)	22.9	(19.8)	22.0	(11.3)	15.2	(20.9)

***Source:*** Prepared by the authors based on the vector surveillance results. Temperature data are from the National Oceanic and Atmospheric Administration (NOAA) station in Brownsville, Texas.

a Standard deviation.

b The three most common species caught in the surveillance traps were 1) *Culex quinquefasciatus,* which transmits West Nile virus (WNV) and Saint Louis encephalitis; 2) *Ae aegypti,* which transmits dengue virus (DENV) and yellow fever virus; and 3) *Ae.Albopictus,* which transmits chikungunya virus, Eastern equine encephalitis virus, WNV, and DENV. However, because so few *Ae. albopictus* mosquitoes were found, they were combined with *Ae. aegypti*to form a new, third category (“*Aedes*spp.”) for the analysis of species incidence over time.

### Vector activity

In 2012, Texas experienced its largest outbreak of WNV. Brownsville had very few human cases, and until recently, that was the only year that any local mosquitoes positive for WNV were recorded. Three years later, in 2015, two more pools of mosquitoes tested positive for WNV. Both specimen pools were from the neighborhood that had WNV-positive mosquitoes in 2012, a finding with interesting public health and preventative implications.

### Vector ecology

Using the mosquito trap results, plus weather and species data from the NOAA, the surveillance teams generated descriptive statistics by year of data collection, average rainfall, and maximum temperature (Table 2). Using Stata statistical software (version 12) (StataCorp LLC, College Station, Texas), a robust regression analysis was conducted to predict the association between mosquito species population patterns, adjusting for year, month, rainfall, and high temperature. Relationships were considered to be significant if the *P* value was < 0.05. Given the history of DENV and WNV in South Texas, and the potential for autochthonous transmission of CHIKV in the region, the surveillance study focused on the vectors that circulate those three diseases. Regression analysis revealed a negative correlation between *C. quinquefasciatus* and month of the year and a positive correlation between *Ae. aegypti* and month of the year. Although the TDSHS in Austin considers the arbovirus season in South Texas to be finished in November, the surveillance teams found large numbers of mosquitoes in the months of December and January. Overall mosquito abundance decreased toward the end of the year, but *Ae. aegypti* mosquitoes continued to survive well into the months traditionally considered post-mosquito season (17). Also, while there seemed to be no significant relationship between average rainfall and mosquito species, there was a correlation between temperature highs and mosquito species.

## DISCUSSION

Based on these analyses, the research team submitted recommendations to the City of Brownsville Public Health Department to guide and refine future surveillance and vector control strategies. Improving the surveillance strategy would allow Brownsville to more proactively mitigate arboviral risk in the community. Ongoing systematic collection of mosquito surveillance data helps establish associations between temporal and weather-related variables and mosquito abundance and increases understanding of the arboviral season. Based on the overall mosquito density and virus activity, the results of this study suggest that Brownsville is currently at high risk for DENV, WNV, and potentially CHIKV (9, 18). In addition, based on the temporal and weather-related data, Brownsville appears to be at risk of arboviral transmission year-round. While other studies have found a correlation between mosquito abundance and rainfall, this study did not. However, this study did find a positive correlation between mosquito species abundance and both temperature highs and month of the year. These results, based on the longitudinal surveillance, could be used to identify and target higher-risk neighborhoods and thus facilitate more efficient allocation of the city’s limited resources by directing public health outreach efforts where they are needed most.

As arboviral threats become more numerous in South Texas, it is vital to prioritize mosquito control programs and support surveillance capacity in areas of highest risk. This includes bolstering entomologic surveillance, enhancing local laboratory infrastructure to increase mosquito and human testing capacity, and al-lotting resources for community education focused on prevention. Vector surveillance helps the City of Brownsville Public Health Department 1) detect the presence of various mosquito species and arboviruses that affect human health; 2) understand vector and disease dynamics over time for better risk assessment; and 3) evaluate the impact of vector control measures and public outreach. All of these data combined can provide better understanding of the local arbovirus epidemic potential (11, 19).

### Limitations

The limitations of this study are largely related to a lack of local funding and resources to support adequate arboviral surveillance in the border regions. The City of Brownsville only employs one or two part-time personnel to conduct weekly surveillance activities. Furthermore, the number of mosquito traps available for use has steadily declined since 2009, as traps have broken but have not been replaced due to lack of funding. On the other hand, the use of traps presented another potential limitation. When looking at species and species abundance, the trap itself can be a study confounder if, due to the design or other characteristics, including location, it attracts only certain species of mosquitoes. For example, almost all sites sampled were in urban areas, which could have contributed to a bias in the species caught.

### Conclusions

Through better understanding of seasonality and areas of mosquito density, public health outreach and community education in high-risk areas can be prioritized. The City of Brownsville Public Health Department’s vector surveillance study represents the first step in unifying arbovirus surveillance systems in South Texas and may serve as a model for similar improvements in other border areas susceptible to arbovirus epidemics.

#### Acknowledgments.

The authors would like to thank the City of Brownsville Department of Public Health, the Kleberg Medical Research Scholars Foundation, and the University of Texas Health Science Center at San Antonio (UTHSCSA) Center for Medical Humanities and Ethics for lending their time, resources, and expertise.

#### Disclaimer.

Authors hold sole responsibility for the views expressed in the manuscript, which may not necessarily reflect the opinion or policy of the *RPSP/PAJPH* or the Pan American Health Organization (PAHO).
